# Whole genome sequencing data of *Candida krusei*, the pathogen causing Candidaemia, from Department of Parasitology Culture Collection, Faculty of Medicine Universitas Indonesia

**DOI:** 10.12688/f1000research.121402.2

**Published:** 2023-03-29

**Authors:** Fadilah Fadilah, Robiatul Adawiyah, Retno Wahyuningsih, Anna Rozaliyani, Ayu Eka Fatril, Rafika Indah Paramita, Linda Erlina, Khaerunissa Anbar Istiadi

**Affiliations:** 1 Department of Medical Chemistry, , Faculty of Medicine, Universitas Indonesia,, Jakarta, Indonesia, 10430, Indonesia; 2 Bioinformatics Core Facilities, Indonesian Medical Education and Research Institute (IMERI), Faculty of Medicine, Universitas Indonesia, Jakarta, Indonesia, 10430, Indonesia; 3 Department of Parasitology, Faculty of Medicine Universitas Indonesia, Jakarta, 10430, Indonesia; 4 Clinical Parasitology Study Programme, Faculty of Medicine, Universitas Indonesia, Jakarta, 10430, Indonesia; 5 Department of Parasitology, School of Medicine, Indonesian Christian University, Jakarta, 13630, Indonesia; 6 Pulmonary Mycosis Centre, Jakarta, 10430, Indonesia

**Keywords:** Candida krusei, fluconazole, genomic profiling, resistance gene

## Abstract

*Candida krusei* is a *Candida* non-albicans species with a high prevalence, which causes candidaemia. Current treatment guidelines include fluconazole as a primary therapeutic option for the treatment of these infections; however, it is only a fungistatic against *Candida spp*., and both inherent and acquired resistance to fluconazole have been reported. *C*. *krusei* species is also reported as the only *Candida sp*. which has an intrinsic resistance factor to fluconazole. Therefore, in dealing with antifungal resistance, it is necessary to develop new antifungal agents that are efficient in the treatment of fungal infections, especially those caused by *C. krusei*. The purpose of this study was to investigate the genome of clinical *C. krusei* isolates and correlate the resistant phenotypes with mutations in resistance genes. A total of 16 samples of *C. krusei* from clinical samples from hospitals in Jakarta were used in the experiment. All colonies were extracted using the QIAamp DNA Mini Kit. The library was prepared using the Illumina DNA Prep Kit. The sequencing process was carried out on the Illumina MiSeq Platform using a 2x301 paired-end configuration. FASTQ raw files are available under the BioProject Accession Number PRJNA819536 and Sequence Read Archive Accession Numbers SRR18739949 and SRR18739964.

## Introduction

*Candida krusei* (teleomorph *Pichia kudriavzevii*) is an opportunistic fungal pathogen. ^1^ It is the fourth most common non-albicans *Candida* species (NAC) causing high prevalence and invasive candidiasis and candidemia. ^2^^,^^3^ *C. krusei* is among unique species with natural resistance towards fluconazole, a commonly used antifungal for *Candida* infections. ^4^ Therefore, in dealing with antifungal resistance, it is necessary to develop new antifungal agents that are efficient in the treatment of fungal infections, especially those caused by *C. krusei* One approach in examining the drug resistance of *C. krusei* is by sequencing the genome with potential roles in resistance. Here we report the whole genome sequencing data of *Candida krusei* from Department of Parasitology Culture Collection, Faculty of Medicine Universitas Indonesia. The sequencing data obtained from this study will help to understand the correlation between phenotypes with mutations in resistance genes and the development of appropriate treatments and medication for the infection.

## Methods

### Sample collection and DNA extraction

The *C. krusei* clinical sample were retrieved from the Department of Parasitology Culture Collection, Faculty of Medicine, Universitas Indonesia. DNA was extracted using the QIAamp DNA Mini Kit (Qiagen, catalog number: 51304) with bead-beating methods. ^5^ 100 μL culture of *C. krusei* were added into 1.5 ml microcentrifuge tube containing 200 μL ATL buffer (Qiagen). Three spoon full of sterilized sand were added into the mixture for bead-beating, which was performed for 10 minutes. 20 μL Proteinase K (Qiagen) were added and the mixture was incubated at 56°C until completely lysed (three hours), and agitated every 10 minutes during incubation time, until homogeneous. 200 μL Buffer AL (Qiagen) were added and the mixture was incubated at 70°C for 10 minutes. 200 μL pure ethanol were added and the mixture was transferred into the QIAamp Mini spin column (Qiagen). Column were centrifugated at 6000 × g for one minute and the flow-through were discarded. QIAamp Mini spin columns were placed innew 2 mL collection tubes and 500 μL Buffer AW1 were added into the column. Columns were centrifugated at 6000 × g for one minute and the flow-through was discarded. QIAamp Mini spin columns were placed into new 2 mL collection tubes and 500 μL Buffer AW2 (Qiagen) were added. Columns were centrifugated at 17000 × g for three minutes and the flow-through was discarded. QIAamp Mini spin columns were transferred into new 1.5 mL microcentrifuge tubes, and 200 μl distilled water were added. Columns were incubated at room temperature for one minute and centrifugated at 6000 × g for one minute to elute the DNA. The quality of extracted DNA (A260/280) was measured using a spectrophotometer (NanoDrop ND-1000). The quantity of extracted DNA was measured with Qubit 4.0 Fluorometer using the dsDNA HS Assay kit.

### Library preparation

DNA libraries were prepared using the Illumina DNA Preparation Kit. The index used for the library preparation was Integrated DNA Technologies, Inc (IDT) for Illumina Nextera Indexes for ligation step. The library construction steps were as follows:

*Tagmentation*

100 ng DNA were added into a 96-well plate and mixed with tagmentation master mix from Illumina Nextera Kit (Illumina). The mix were then incubated at 55°C in a thermal cycler (The Applied Biosystems ProFlex PCR System) for 15 minutes.

*Post tagmentation clean-up and amplification of tagmented DNA*

Illumina Nextera Kit tagment stop buffer (Illumina) were added to the tagmentation reaction and incubated at 37°C for 15 minutes. The mixture was washed with Illumina Nextera Tagment wash buffer (Illumina) on the magnetic stand. The tagment Wash Buffer was discarded and Nextera PCR master mixes (Illumina) were added onto the beads. Index adapters were added as sample barcoding. The mixture was amplified in thermal cycler.

*Libraries clean-up*

The beads were added into the supernatant of the mixture and washed twice using 80% ethanol on a magnetic stand. Nextera resuspension buffer (Illumina) reagents were added onto beads and final libraries were retrieved from the supernatant. The libraries of each sample were pooled. ^6^

### Whole genome sequencing

The barcoded DNA libraries were sequenced using an Illumina Miseq Platform on the v3-Flow Cell. The DNA library was denatured according to the manufacturer’s protocol. ^7^ For quality control, the library was spiked with 1% PhiX Control v3. The sequencing run produced 2 × 301 bp paired-end libraries. The data were deposited to the NCBI Sequence Read Archive (SRA) under BioProject. Total raw reads were obtained using FastQC software, and the total raw bases and percentage of Q30 were evaluated using q30 python scripts. ^8^

### Ethical approval

This research was approved by the Faculty of Medicine Universitas Indonesia Ethical Committee (approval number: 970/UN2.F1/ETIK/PPM.00.02/2021).

## Results

The whole raw genome sequence data of 16 clinical isolates of *C. krusei* from the Department of Parasitology, Faculty of Medicine of Universitas Indonesia were deposited into NCBI data under BioProject accession number PRJNA819536 and SRA SRR18739949-SRR18739964. The DNA quality and quantity of the samples are shown in Table 1. Information regarding the raw data is described in Table 2. These raw data of *C. krusei* genome are useful for genome profiling and correlating the resistant phenotypes with mutations in resistance genes.

**Table 1.** DNA quantity and quality.

Sample	Concentration (ng/μL)	Purity (A260/A280)
CK1_2021	13.4	2.1
CK2_2021	39.3	2.03
CK3_2021	42.3	2.08
CK4_2021	38.3	2.02
CK5_2021	34.3	1.95
CK6_2021	45.4	1.93
CK7_2021	37	2.07
CK8_2021	23	2.03
CK9_2021	34.3	2.09
CK10_2021	16.7	2.1
CK11_2021	18.9	2.05
CK12_2021	10.3	1.91
CK13_2021	14.2	1.97
CK14_2021	10.1	1.86
CK15_2021	17.9	2
CK16_2021	8.95	1.98

**Table 2.** Information of raw sequencing data of *C. krusei* clinical isolates.

Sample	BioSample accession number	SRA accession number	Q30 (%)	GC content (%)
CK1_2021	SAMN26901813	SRR18739964	89.75	36
CK2_2021	SAMN26901814	SRR18739963	88.98	38
CK3_2021	SAMN26901815	SRR18739956	89.66	38
CK4_2021	SAMN26901816	SRR18739955	90.00	37
CK5_2021	SAMN26901817	SRR18739954	88.83	37
CK6_2021	SAMN26901818	SRR18739953	89.67	37
CK7_2021	SAMN26901819	SRR18739952	89.57	38
CK8_2021	SAMN26901820	SRR18739951	90.40	37
CK9_2021	SAMN26901821	SRR18739950	92.39	37
CK10_2021	SAMN26901822	SRR18739949	89.34	37
CK11_2021	SAMN26901823	SRR18739962	90.65	37
CK12_2021	SAMN26901824	SRR18739961	90.42	37
CK13_2021	SAMN26901825	SRR18739960	93.18	34
CK14_2021	SAMN26901826	SRR18739959	89.01	37
CK15_2021	SAMN26901827	SRR18739958	88.69	37
CK16_2021	SAMN26901828	SRR18739957	89.57	37

## Data availability

### Underlying data

Raw data (FASTQ) files have been deposited into National Center for Biotechnology Information (NCBI, https://www.ncbi.nlm.nih.gov).

BioProject: Raw sequence reads of *Candida krusei* from clinical samples, Accession Number: PRJNA819536; https://www.ncbi.nlm.nih.gov/bioproject/PRJNA819536

BioSample: Pathogen: clinical or host-associated sample from Pichia kudriavzevii, Accession Number: SAMN26901813; https://identifiers.org/ncbiprotein:SAMN26901813

BioSample: Pathogen: clinical or host-associated sample from Pichia kudriavzevii, Accession Number: SAMN26901828; https://identifiers.org/ncbiprotein:SAMN26901828

Raw sequence reads of *Candida krusei* from clinical samples:

Sequence Read Archive: Raw sequence reads of Candida krusei from clinical samples, Accession Number: SRR18739949; https://identifiers.org/insdc.sra:SRR18739949

Sequence Read Archive: Raw sequence reads of Candida krusei from clinical samples, Accession Number: SRR18739964; https://identifiers.org/insdc.sra:SRR18739964
