# Supplementary material for: Whole genome sequencing data of Candida krusei, the pathogen causing Candidaemia, from Department of Parasitology Culture Collection, Faculty of Medicine Universitas Indonesia
Source: F1000Res. 2023 Mar 29;11:593. Originally published 2022 Jun 1. [Version 2] doi: 10.12688/f1000research.121402.2 (PMC10320322; doi:10.12688/f1000research.121402.2)
Supplement: Peer review history

# Peer review: Reviewer response for version 2

**DOI:** 10.5256/f1000research.143748.r168114

**Published:** 2023-07-04

**Authors:** Atanu Banerjee

**Affiliations:** 1 Amity Institute of Biotechnology,, Amity University Haryana, Manesar, Haryana, India

No further comments.

Are the datasets clearly presented in a usable and accessible format, and the assembly and annotation available in an appropriate subject-specific repository?

Partly

Are sufficient details of the sequencing and extraction, software used, and materials provided to allow replication by others?

Yes

Are the rationale for sequencing the genome and the species significance clearly described?

Partly

Are the protocols appropriate and is the work technically sound?

Yes

Reviewer Expertise:

Mycology. Antifungal resistance, Candida species, Efflux pumps, structure-function studies, sequence, and structural bioinformatics

I confirm that I have read this submission and believe that I have an appropriate level of expertise to confirm that it is of an acceptable scientific standard.

# Peer review: Reviewer response for version 1

**DOI:** 10.5256/f1000research.133265.r159413

**Published:** 2023-01-09

**Authors:** Atanu Banerjee

**Affiliations:** 1 Amity Institute of Biotechnology,, Amity University Haryana, Manesar, Haryana, India

The article by Fadilah et al, presents the whole genome sequence data of clinical isolates of *C. krusei*. While the genome sequence data generated could be useful to advance our knowledge regarding factors that might be responsible for the intrinsic azole resistance exhibited quite often by *C. krusei* isolates, there are some significant gaps that need to be filled to make the manuscript more useful for readers. I elaborate on some of them:

1. The introduction seems extremely brief and does not bring the importance of the work into the proper context. For instance, the authors write " *Therefore, in dealing with antifungal resistance, it is necessary to develop new antifungal agents that are efficient in the treatment of fungal infections, especially those caused by C. krusei"  *and then directly mention " *The purpose of this study was to investigate the genome of clinical isolates of C. krusei and correlate the resistant phenotypes with mutations in resistance genes.". *The two statements do not bring the perspective and importance into place appropriately. 
  Also, there isn't much background literature provided in this section.
2. The authors mention non- *albicans* *Candida* (NAC) species incorrectly in the abstract and introduction. It should be corrected.
3. The authors do not provide any background information related to the clinical isolates, except the center. It is extremely important to at least briefly provide some background information on the isolates.
4. While the authors state that "The purpose of this study was to investigate the genome of clinical isolates of *C. krusei* and correlate the resistant phenotypes with mutations in resistance genes", there is no information provided regarding the same. It limits the importance of the paper.
5. In the keywords, " *Candida krusei*" is non-italicized. "Resistance gene" does not make much sense and should be replaced with something more meaningful. The same should also be replaced elsewhere in the MS.

Are the datasets clearly presented in a usable and accessible format, and the assembly and annotation available in an appropriate subject-specific repository?

Partly

Are sufficient details of the sequencing and extraction, software used, and materials provided to allow replication by others?

Yes

Are the rationale for sequencing the genome and the species significance clearly described?

Partly

Are the protocols appropriate and is the work technically sound?

Yes

Reviewer Expertise:

Mycology. Antifungal resistance, Candida species, Efflux pumps, structure-function studies, sequence, and structural bioinformatics

I confirm that I have read this submission and believe that I have an appropriate level of expertise to confirm that it is of an acceptable scientific standard, however I have significant reservations, as outlined above.

# Peer review: Reviewer response for version 1

**DOI:** 10.5256/f1000research.133265.r147383

**Published:** 2022-08-30

**Authors:** Xuepeng Sun

**Affiliations:** 1 Collaborative Innovation Center for Efficient and Green Production of Agriculture in Mountainous Areas of Zhejiang Province, College of Horticulture Science, Zhejiang A&F University, Hangzhou, China

Drug resistance is a serious problem for human disease. As the human pathogen *C. krusei* has natural resistance to the commonly used azole fungicides, it is of particular importance to explore the molecular basis underlying the resistance. This study provides high-depth sequence data for 16 clinic isolates of *C. krusei*, which in combination with published *C. krusei* genome will provides new information for correlating the mutations with the resistance phenotypes. Due to the potential interest of the data to scientific community and the quality of the manuscript, I would suggest an acceptance of this manuscript in F1000Reseach.

Are the datasets clearly presented in a usable and accessible format, and the assembly and annotation available in an appropriate subject-specific repository?

Yes

Are sufficient details of the sequencing and extraction, software used, and materials provided to allow replication by others?

Yes

Are the rationale for sequencing the genome and the species significance clearly described?

Yes

Are the protocols appropriate and is the work technically sound?

Yes

Reviewer Expertise:

Genomics

I confirm that I have read this submission and believe that I have an appropriate level of expertise to confirm that it is of an acceptable scientific standard.
